# Silibinin as a promising treatment for diabetes: Insights into behavioral and metabolic changes in an animal model

**DOI:** 10.1002/fsn3.3999

**Published:** 2024-02-09

**Authors:** Asli San Dagli Gul, Gulbahar Boyuk Ozcan, Okan Arihan

**Affiliations:** ^1^ Department of Physiology, Faculty of Medicine Hacettepe University Ankara Turkey; ^2^ Department of Physiology, Faculty of Medicine Ankara Medipol University Ankara Turkey

**Keywords:** behavior, diabetes mellitus, insulin, silibinin, streptozotocin

## Abstract

Diabetes mellitus is causing serious health problems in the chronic period. Silibinin is a flavonoid obtained from the milk thistle (*Silybum marianum*), which is among the herbal ethnopharmacological administrations. In studies with silibinin, it has been reported that it increases the activity of pancreatic beta cells and insulin sensitivity and has a hyperglycemia‐reducing effect. However, behavioral parameters have not been evaluated together with insulin levels and liver function tests. Our aim in this study was to examine the effects of silibinin on insulin secretion, anxiety‐like behaviors, and learning in a streptozotocin (STZ)‐induced rat diabetes model. Wistar albino rats weighing 200–250 g were divided into 4 groups. Control: Saline solution, Diabetes: STZ 45 mg/kg, S 100: STZ 45 mg/kg + Silibinin 100 mg/kg, S 200: STZ 45 mg/kg + Silibinin 200 mg/kg. Administrations were continued for 21 days. On the 21st day, open field and elevated plus maze as unconditional anxiety tests; Barnes maze for learning and memory; and rotarod test for locomotor activity were conducted. Following behavioral tests, blood samples were taken under anesthesia. Blood glucose levels and ALT values were measured. Insulin levels were measured with an ELISA plate reader. Silibinin shortened the time to find the correct hole. Silibinin prevented the decrease in insulin due to STZ, exhibited a hyperglycemia‐reducing effect and decreased the elevation of ALT.

## Graphical abstract

Silibinin is a flavonoid obtained from the milk thistle (*Silybum marianum*), which is among the herbal ethnopharmacological administrations. Silibinin reversed the STZ‐induced locomotor activity decrease observed in the rotarod test. It prevented the decrease in insulin due to STZ, exhibited a hyperglycemia‐reducing effect, and decreased the elevation of ALT.

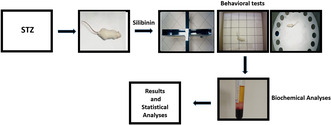

## 1 INTRODUCTION

### 1.1 Blood glucose and diabetes

Numerous hormones, such as growth hormone, insulin, glucagon, cortisol, and thyroid hormones, play a role in regulating blood glucose. However, the primary hormone responsible for facilitating the uptake of glucose into cells when blood glucose levels rise is insulin. Diabetes mellitus is a chronic condition characterized by elevated blood glucose levels resulting from insufficient insulin production or impaired insulin utilization in the body. This condition leads to heterogeneous metabolic issues (World Health Organization, 2019).

In cases of insulin deficiency, treatment options include insulin secretagogues and replacement therapies when there is a complete absence of insulin. On the other hand, for peripheral insulin resistance, medications aimed at overcoming this resistance are used. When exploring treatment alternatives using ethnopharmacological substances, it has been determined that over 1200 plant species are used worldwide for diabetes treatment (Jugran et al., 2021). Among these herbal remedies, silibinin, an active flavonolignan, is derived from milk thistle (*Silybum marianum*) (Fanoudi et al., 2020). *Silybum marianum* contains various active compounds, including silibinin, silychristin, quercetin, silydianin, and taxifolin. While there are different isoforms of silibinin, it has been reported that after oral administration, silibinin is absorbed from the entire gastrointestinal system, especially the duodenum, at different levels and under varying acidic conditions (Tomou et al., 2023).

This plant, used to increase milk production in breastfeeding mothers, is also administered for the treatment of gallbladder and liver diseases due to its antioxidant and hepatoprotective properties. Studies involving silibinin have reported corrective effects on diabetic hyperglycemia by enhancing the activity of pancreatic beta cells, increasing insulin sensitivity in liver and muscle cells, and reducing lipid accumulation in adipocytes (Suh et al., 2015). Additionally, silibinin has been found effective in treating various diabetic complications, including neuropathy, retinopathy, impaired wound healing, hepatopathy, cardiomyopathy, nephropathy, and osteoporosis (Chu et al., 2018). It appears that these effects of silibinin stem from its antioxidant and anti‐inflammatory properties.

Due to the poor content of antioxidant enzymes, pancreatic Langerhans cells are vulnerable to oxidative stress. Accumulated intracellular reactive oxygen species (ROS) leads to increased beta cell apoptosis and reduced glucose sensing (Lenzen, 2017). Additionally, it affects beta cell functions by disrupting glucose‐stimulated insulin gene expression and insulin secretion (Lytrivi et al., 2020). Hyperglycemia, along with dyslipidemia and inflammation, contributes to excessive ROS formation, and agents targeting these mechanisms reduce diabetic complications (Khater et al., 2022). Silibinin (10 μM) has been demonstrated to mitigate oxidative damage caused by high glucose levels (30 mM glucose) (Palomino et al., 2017).

In a study conducted with obese db/db mice, silibinin was shown to reduce insulin resistance and prevent myocardial and hepatic damage (Salamone et al., 2012). There are numerous studies regarding the anti‐diabetic and neuroprotective effects of silibinin. In addition to animal studies, research involving silibinin on topics such as diabetes and dementia has been conducted in humans as well (Fernandes et al., 2018; Marrazzo et al., 2011).

Diabetes is not only responsible for the aforementioned issues but also contributes to behavioral problems. In the investigation of these problems using animal models, various parameters such as learning, memory, and anxiety are examined. The effects of diabetes on these parameters and the protective effects of potential therapeutic agents are tested. One of the models used for this purpose is the Barnes maze, which evaluates spatial learning strategies and memory capacity in rodents such as mice and rats. It serves as a laboratory test that assists in studying the effects of neurological disorders. On a circular platform with holes, one hole leads to a safe zone, while the other holes are misleading. Animals are trained to learn and locate the target hole at varying durations and are subsequently tested by removing the safe zone (Harrison et al., 2006).

Another component of these behavioral studies is anxiety. Anxiety can be described as a physiological, psychological, and behavioral state that arises from the impact of real or potential threats on the survival of organisms (Steimer, 2002). In a meta‐analysis examining whether diabetes is related to anxiety, data from 12,626 individuals were reviewed, and a positive and significant correlation was found between the presence of anxiety disorders or symptoms and diabetes (Smith et al., 2013).

Experimental anxiety models include various methods such as elevated plus maze (EPM), open field test (OFT), Light/Dark Box Test, Social Isolation Test, Forced Swim Test, Exposure Tests, and Fear Conditioning Test (Steimer, 2022). It is recommended to use different test sequences for various anxiety types and different animal species in anxiety studies. Some of the tests, like EPM, open field, and light/dark box, can be used together (van Gaalen & Steckler, 2000).

Given the diversity of the tests mentioned above, it's evident that there are numerous experimental models for investigating various disease conditions or disorders triggered by chemical agents. While the literature highlights many positive effects of silibinin, particularly on diabetic complications, its impacts on both behavior and locomotor activity in diabetic rats have not been adequately explored. Therefore, the aim of this study is to investigate whether silibinin will not only have a regulatory effect on blood glucose levels in diabetic rats but also potentially induce positive behavioral changes that are hampered by experimental DM.

## 2 MATERIALS AND METHODS

In this study, a total of 40 Wistar albino rats weighing between 200 and 250 g were included (4 groups). Throughout the experimental procedure, rats were given ad libitum access to water and food, and standard housing conditions were maintained at 20 ± 2°C with a 12‐h light/dark cycle. After a sufficient adaptation period, rats were fasted overnight and, except for the control group, administered a STZ injection (45 mg/kg) to induce diabetes. Blood glucose levels were measured via tail using a glucometer 72 h after injection of STZ to monitor diabetes development, and animals with blood glucose levels of 300 mg/dL and above were selected and sorted to different groups (*n* = 8, except the S100 group, which has *n* = 6 animals) (Table 1). Permission from the local ethical committee was obtained prior to the study (2020/03‐0).

**TABLE 1:** Experimental groups.

Groups	Administration
Control	Saline solution
Diabetes	Streptozotocin 45 mg/kg
S100	Streptozotocin 45 mg/kg + Silibinin 100 mg/kg
S200	Streptozotocin 45 mg/kg + Silibinin 200 mg/kg

Silibinin was administered to the S 100 and S 200 groups via oral gavage at the mentioned doses (100–200 mg/kg) for a duration of 21 days, with a single dose per day. Serum glucose levels were studied through biochemical laboratory analysis of blood obtained under anesthesia at the end of the experiment.

Upon completion of the experimental protocol, behavioral tests were initiated. No animals were excluded from the experiment due to death or complications during the experimental period. The experimental procedures adhered to the required ethical standards and animal welfare regulations, and at the end of the experiment, the animals were sacrificed under ketamine/xylazine anesthesia to obtain blood samples.

On the final day of the experiment, the following tests were conducted to evaluate various aspects of behavior and cognitive function:

Anxiety Assessment: OFT and EPM were employed to assess unconditional anxiety levels.
Cognitive Tests: The Barnes maze was employed to evaluate spatial memory. This test was carried out on the fourth day, following 3 days of training.
Locomotor Activity Assessment: Rotarod test was conducted to evaluate locomotor activity. Data collection took place after the rats underwent preliminary training. In the rotarod test, the rats' latency times (duration of remaining on the rotating rod) were recorded as the device rotated at 16 rpm.

Subsequent to all behavioral tests, blood samples were collected under ketamine/xylazine anesthesia. After centrifugation, the supernatants were collected, and blood glucose and ALT/AST levels were assessed in the biochemical laboratory. Serum insulin levels were measured using an ELISA kit, and the resulting curve was interpreted.

Statistical analysis was performed using the GraphPad Prism 8 software. Following one‐way ANOVA variance analysis for the groups, group comparisons were carried out using the Tukey test. The results are expressed as means ± standard errors of the mean, and differences with *p*‐values less than .05 were considered statistically significant.

## 3 RESULTS

### 3.1 Behavioral tests

#### 3.1.1 EPM

**FIGURE 1:**
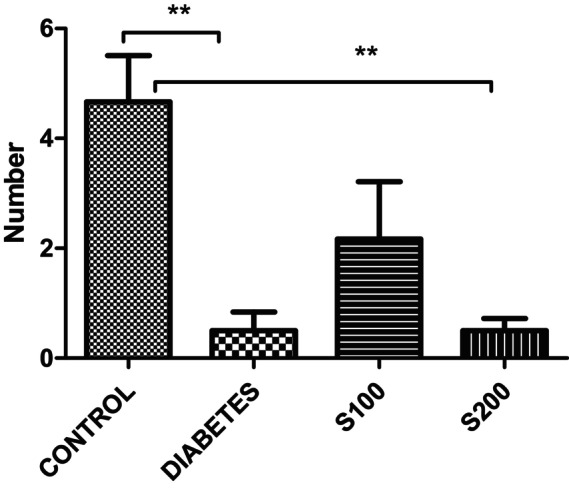
Results of the elevated plus maze. Number of entries into the open arm (**p < .01).

**FIGURE 2:**
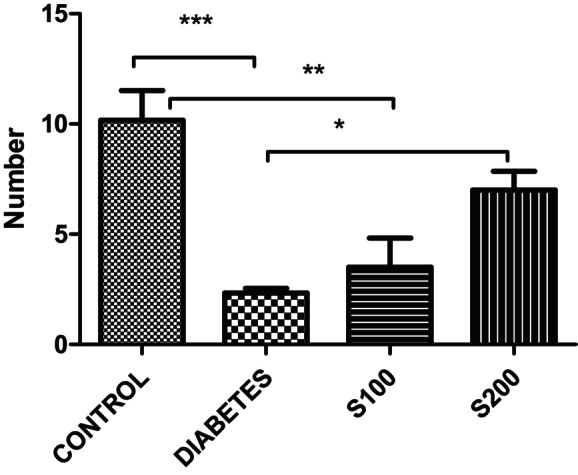
Results of the elevated plus maze. Number of entries into the closed arm (*p < .05, **p < .01, and ***p < .001).

The results from the EPM reveal that in the control group, the number of entries into the open arm (Figure 1) and the number of entries into the closed arm (Figure 2) were significantly higher compared to diabetes group (*p* < .05).

**FIGURE 3:**
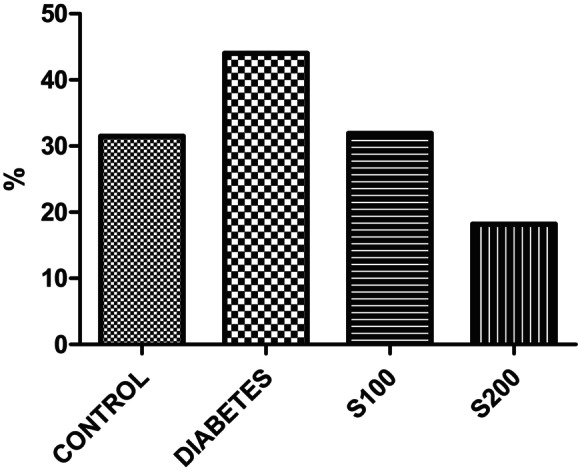
Results of the elevated plus maze. Closed arm/open arm entry ratio (*p* > .05).

However, to associate this activity with anxiety, the ratio of entries into the closed arm to entries into the open arm provides more insight (Figure 3). When examining the closed arm entry/open arm entry ratio, it appears that the diabetes group has a higher ratio and the S200 group has a lower ratio, but statistical significance was not found.

#### 3.1.2 Rotarod test

**FIGURE 4:**
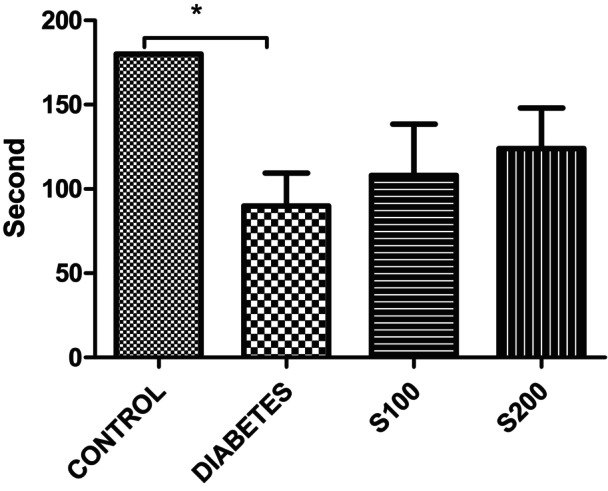
Results of the Rotarod Locomotor Activity Test (*p < .05).

In the rotarod test, where rats were subjected to 3 sessions of 60 s each (for a total of 180 s), the control group exhibited the highest performance, while the diabetes group displayed the lowest performance (Figure 4). The difference between the control and diabetes groups (*p* < .01) is statistically significant.

#### 3.1.3 Barnes maze

**FIGURE 5:**
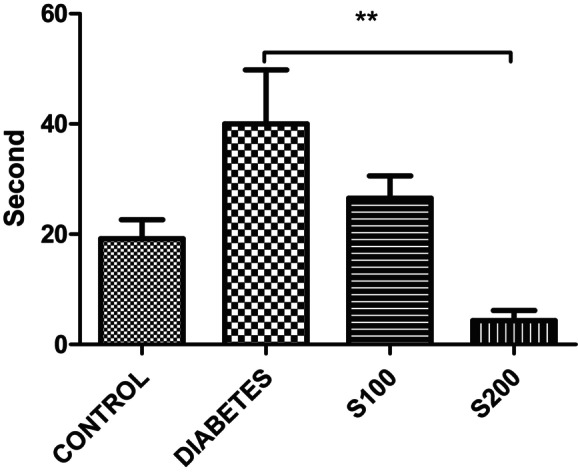
Results of the Barnes maze. Time to find correct escape hole (**p < .01).

In the Barnes maze, the diabetes group took a significantly longer time to find the escape box compared to the S200 group (*p* < .01) (Figure 5). However, no significant difference was observed between the silibinin‐treated groups and the control group. The S200 group effectively reversed the negative impact caused by STZ (*p* < .01).

#### 3.1.4 OFT

**FIGURE 6:**
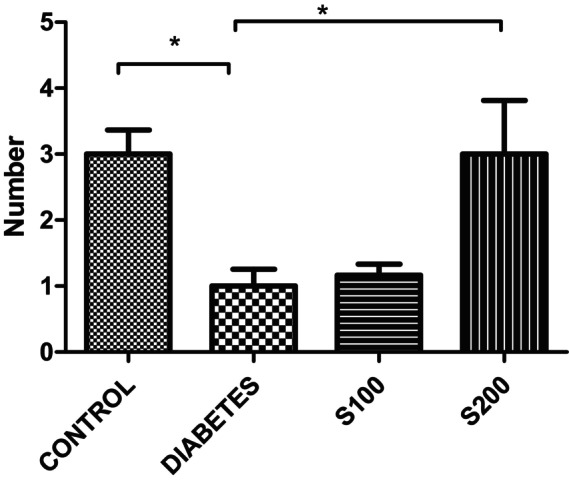
Number of entries into the central area in the open field test (*p < .05).

In the OFT, a decrease in the number of entries into the central area was observed in the diabetes and S100 groups compared to the control group. However, statistical significance was observed only between the control and diabetes groups, as well as between the diabetes and S200 groups (*p* < .05) (Figure 6).

**FIGURE 7:**
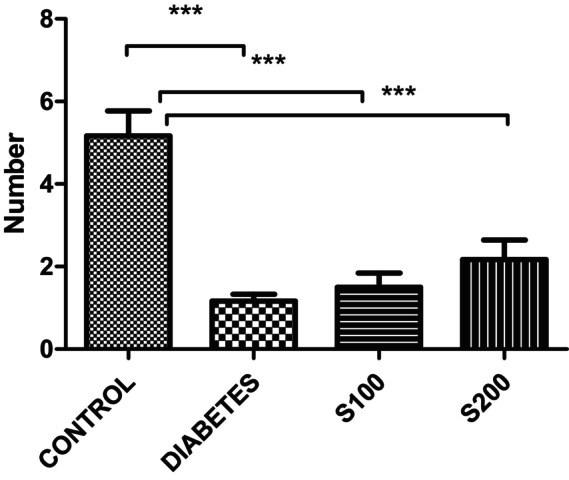
Number of entries into the peripheral areas in the open field test (***p < .001).

The number of entries into the peripheral areas in both the diabetes and silibinin groups was significantly lower compared to the control group (*p* < .01) (Figure 7).

**FIGURE 8:**
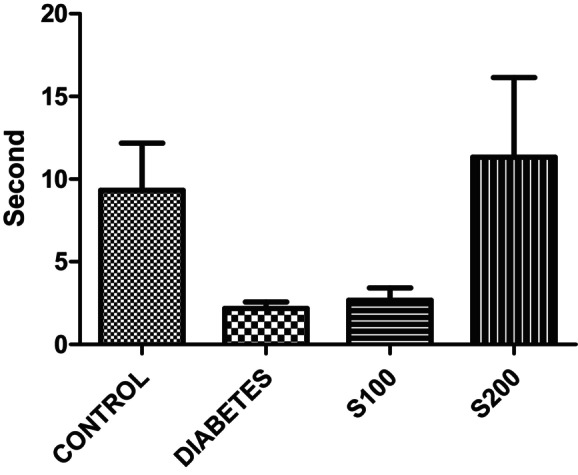
Time spent in the central area by rats in the open field test (*p* > .05).

In the OFT, rats in the control and S200 groups spent more time in the central area; however, the difference was not statistically significant (Figure 8).

### 3.2 Biochemical results

#### 3.2.1 ALT and AST measurements

**FIGURE 9:**
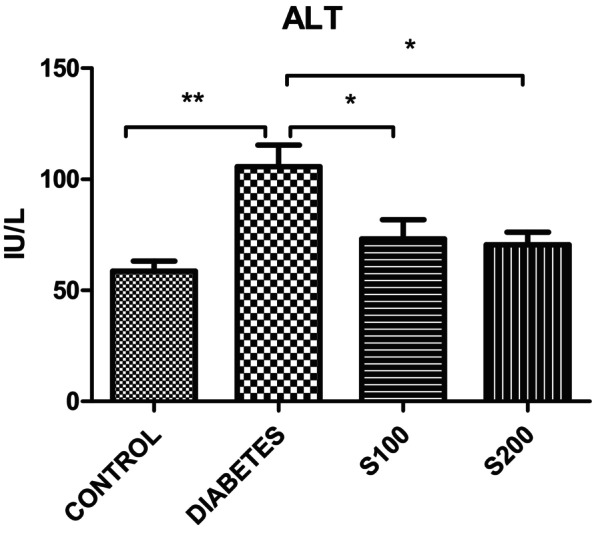
ALT results (*p < .05, **p < .01).

ALT levels, an important marker of liver damage caused by various pathological reasons, were significantly elevated in the diabetes group compared to the control group. Silibinin administration in both doses effectively reduced ALT levels (*p* < .05) (Figure 9).

**FIGURE 10:**
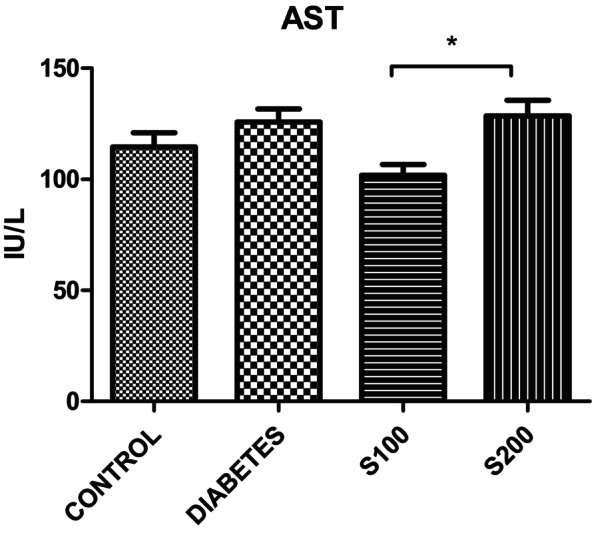
AST results (*p < .05).

The administration of silibinin in the S100 group has reduced AST levels compared to all groups, and this reduction was statistically significant only when compared to S200 (*p* < .05) (Figure 10).

#### 3.2.2 Blood glucose measurements

**FIGURE 11:**
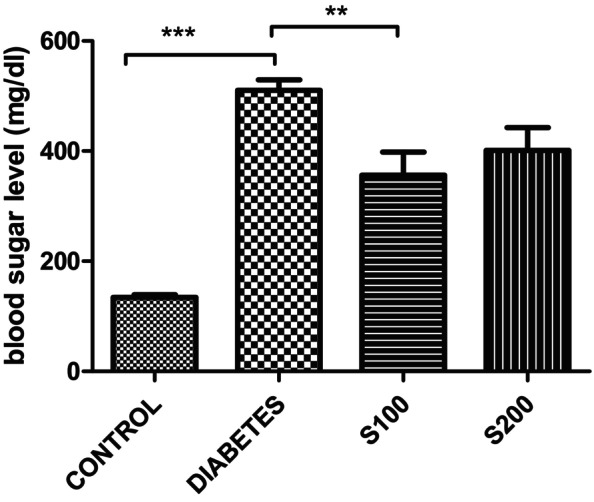
Last day blood glucose values (mg/dL) (***p < .0001, **p < .01).

The blood glucose measurements on the last day indicate that the administration of silibinin decreased the augmented blood glucose levels due to STZ administration (Figure 11). The control group was significantly lower than all groups (*p* < .0001). When the diabetes and S100 groups were compared, the decrease in the S100 group was found to be significant (*p* < .01).

#### 3.2.3 Blood insulin levels

**FIGURE 12:**
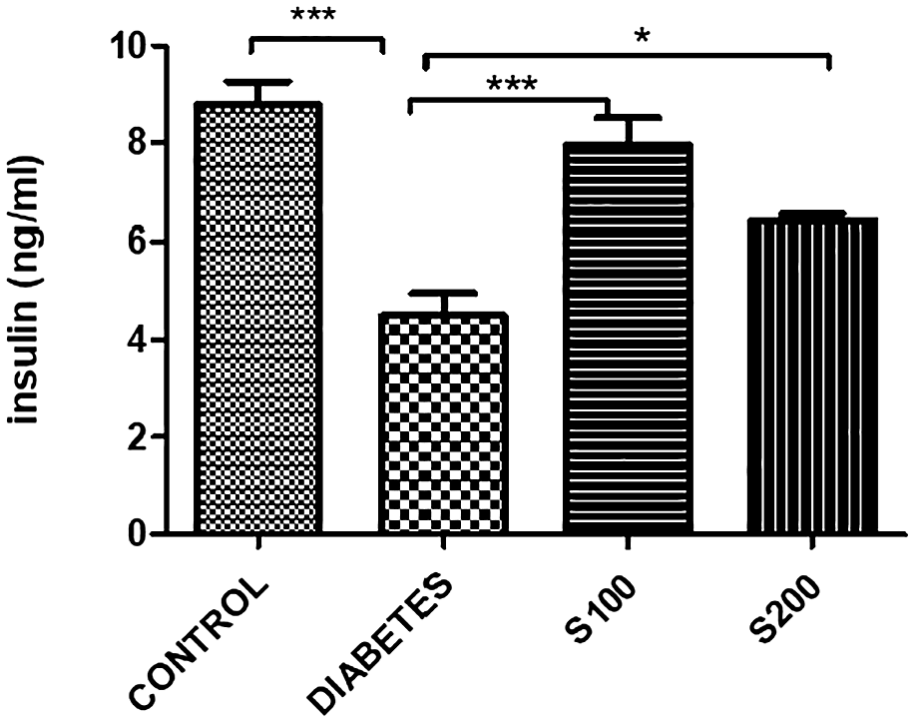
Insulin results in rat blood (*p < .05, ***p < .001).

It was observed that only the diabetes group had decreased insulin levels, and the administration of silibinin in both doses increased insulin levels (*p* < .05) (Figure 12).

## 4 DISCUSSION

Diabetes mellitus is a globally widespread and chronic metabolic disorder that results in consistently high blood glucose levels. The treatment of diabetes aims to control blood glucose levels, reduce insulin resistance, and enhance insulin production by the pancreas. Silibinin, the main active component of milk thistle, possesses various biological effects such as antioxidant, anti‐inflammatory, anti‐diabetic, and liver protective properties. However, there is insufficient research in the literature regarding the effects of antidiabetic properties on behavior and learning.

In this study, we aimed to evaluate the effects of silibinin on anxiety, locomotor activity, spatial memory, and its relationship with liver protective effects in a rat model of diabetes induced by STZ. STZ is a glucose analog that selectively accumulates in pancreatic β‐cells via low‐affinity glucose transporter 2 (GLUT2), leading to cytotoxic effects and commonly used to induce experimental diabetes in rats (Jain et al., 2016; Lenzen, 2008). We conducted different analyses on the effects of silibinin in our rat diabetes model induced by streptozotocin, focusing on hyperglycemia, insulin levels, and cognitive functions, leading to the results mentioned above. The findings we obtained are discussed in detail in the subsections below.

### 4.1 Silibinin decreases diabetic hyperglycemia

The hyperglycemia‐reducing effect of silibinin in the animal diabetes model induced by STZ can be explained by factors such as residual pancreatic function, a direct increase in insulin secretion from Langerhans beta cells, mitigating insulin resistance, or glycemic control through the gut–brain–liver axis.

The reduction of diabetic hyperglycemia in the silibinin‐treated groups may stem from the incomplete destruction of pancreatic beta cells caused by STZ. Although insulin levels were expected to be low in all groups subjected to STZ application (Diabetes, S 100, S 200), a significant decrease was observed only in the diabetes group. The detection of some level of insulin secretion in the diabetes group suggests that the dose (45 mg/kg STZ) we used for inducing pancreatic beta cell damage did not cause complete and absolute destruction but rather indicates the presence of residual surviving cells. However, the significant increase in insulin levels in both silibinin‐treated groups compared to the diabetes group implies that silibinin might enhance beta cell survival and avoid apoptosis, as shown in different studies (Chen et al., 2014; Rahimifard et al., 2018).

Additionally, the anti‐inflammatory and antioxidant effects of silibinin may also play a role in enhancing beta cell survival (Dinić et al., 2022; Yang et al., 2018). Another explanation for the ameliorative effect of hyperglycemia could be due to silibinin's promotion of SIRT‐1 expression, preventing autophagy, and exhibiting a reparative effect on damaged pancreatic cells (Wang et al., 2012). In addition, it is also speculated that silibinin could enhance insulin production and stimulate insulin secretion in pancreatic beta cells. This suggests that silibinin could have potential benefits for controlling diabetes. By appropriately reducing elevated blood glucose levels in diabetes, silibinin prevents the accumulation of glucose inside cells.

Our findings from the last day of serum glucose biochemical measurement particularly indicate a reversive effect of silibinin administration on the hyperglycemic condition induced by STZ (Figure 11).

In a study conducted on RINm5F‐insulinemic cells, silibinin was found to enhance cellular insulin and glucose sensitivity under glucotoxic conditions (Mazraesefidi et al., 2023). Moreover, an in vivo study reported that impaired glucose homeostasis and insulin secretion in C57BL/6J mice subjected to a high‐fat diet were restored by oral administration of 200 mg/kg silibinin for 10 weeks (Chen et al., 2015). A study by Chu et al. (2020) on pancreatic β‐cells under conditions of high glucose and high fat demonstrated that upregulation of estrogen receptor‐α (ERα) expression induced insulin synthesis in pancreatic β‐cells.

Exposure to high glucose levels can lead to pancreatic β‐cell dysfunction, cellular lipid accumulation, cell apoptosis, impaired glucose‐stimulated insulin secretion (GSIS), and potential glucotoxicity. In their article, Chen et al. (2014) mentioned that silibinin, after exposure to high glucose for 24 or 72 h, partially reduced GSIS and increased insulin secretion by regulating β‐cell function through the Insig‐1/SREBP‐1c pathway. In rodents with diabetes induced by STZ, insulin resistance is also commonly observed.

Silibinin might also enhance insulin secretion from pancreatic beta cells, which could assist in controlling blood glucose levels and alleviating the effects of diabetes. Silibinin has been shown to reduce visceral obesity by decreasing visceral fat, upregulating adipose triglyceride lipase (ATGL) expression to enhance lipolysis, downregulating genes such as phosphoenolpyruvate carboxykinase and glucose‐6‐phosphatase to inhibit gluconeogenesis, and increasing the slope of the insulin tolerance test (Yao et al., 2013). Silibinin has been shown to reduce insulin resistance by downregulating GLUT4 translocation in C2C12 myotubes (Li et al., 2015). Another potential explanation for the hypoglycemic effect of silibinin is related to the gut–brain–liver axis (Xu et al., 2018). Colturato et al. (2012) showed the positive effects of silibinin on liver glucose metabolism.

### 4.2 Hepatoprotective effect of silibinin

One of the significant complications of diabetes mellitus is hepatopathy. Diabetes is a risk factor for non‐alcoholic fatty liver disease or glucotoxic liver injury associated with insulin resistance, and silibinin has long been used as a hepatoprotective agent in cases of prolonged hyperglycemia (Dey & Lakshmanan, 2013). Insulin resistance develops in mice with experimental non‐alcoholic steatohepatitis. Silibinin (20 weeks/day IP, daily for 4 weeks) was shown to reduce fasting glucose and insulin levels, reverse insulin resistance, improve liver steatosis by suppressing oxidative stress and NF‐KB activation (Salamone et al., 2012). The combination of silibinin and vitamin E complex has been shown to have therapeutic effects on non‐alcoholic fatty liver disease and improve insulin sensitivity (Lv et al., 2021). The hepatoprotective effect of silibinin also provides a combined benefit by overcoming insulin resistance in diabetes mellitus.

The results of this study are in line with the literature. Among the indicators of liver damage due to various pathological reasons, a significant increase in ALT levels induced by STZ administration was observed compared to the control group. The administration of silibinin was found to reverse this increase (*p* < .05). This situation indicates the liver‐protective effect of silibinin against STZ injection‐induced damage.

### 4.3 Effects of silibinin on behavioral parameters

#### 4.3.1 Rotarod test

The rotarod test is commonly used to measure locomotor activity or assess diseases related to the central nervous system. Liu, Liu, et al. (2021) demonstrated that in a mouse Parkinson's model triggered by MPTP, the walking times of mice were significantly shortened, and the animals fell more quickly, compared to the control group. In the same study, silibinin at doses of 150 and 280 mg/kg significantly improved rotarod test results. In our study, during the rotarod test, the control group exhibited the highest performance, while the diabetes group showed the lowest performance (Figure 4). This situation could be interpreted as STZ administration leading to a decrease in locomotor activity in rats. The S100 and S200 groups showed better performance compared to the diabetes group (*p* > .05), despite a decrease compared to the control group. This data indicate that silibinin administration partially reverses the negative effects caused by STZ injection. According to the study by Lee et al. (2020) rats treated with 100 mg/kg silibinin reversed the decrease in STZ‐induced immobility in the OFT. This finding suggests a similar effect in our study, where the decrease associated with STZ in the rotarod test is reversed through silibinin.

#### 4.3.2 EPM

According to the meta‐analysis study conducted by Amiri and Behnezhad (2019), there is a positive correlation between diabetes and anxiety‐like symptoms. In our study, the EPM showed an increase in the closed arm/open arm ratio compared to the control group due to STZ administration, indicating an increase in anxiety‐like behavior due to STZ.

In our study, the results of the EPM indicate that the number of entries into the open arm (Figure 1) and the closed arm (Figure 2) is significantly higher in the control group compared to all other groups (*p* < .05). This suggests that the control rats are more active than all the other groups. However, the ratio of entries into the closed arm to entries into the open arm provides additional information when correlating this activity with anxiety (Figure 3). When examining the ratio of closed arm entries to open arm entries, all groups appear to have a higher ratio of closed arm entries compared to the control group. Although not statistically significant, this suggests that these groups have a higher level of anxiety, and the increase in anxiety‐like behavior parameters caused by STZ administration is not significantly reduced by S200 administration.

#### 4.3.3 OFT

In this study, the OFT, which is an anxiety test, revealed significant decreases in the number of entries to the central area in the diabetes group compared to the control group, indicating an increase in anxiety (*p* < .05) (Figure 6). This suggests that STZ administration increases anxiety‐like behaviors. In the S200 group, a higher number of entries into the central area were observed compared to the diabetes group. The administration of 200 mg/kg of silibinin significantly reduced this anxiety‐like behavior and exhibited an anxiolytic effect (*p* < .05).

The number of entries to the periphery, a parameter indicating an increase in anxiety, showed a significant decrease in the diabetes group compared to the control group (*p* < .05) (Figure 7). While this could be interpreted as an anxiolytic effect of STZ, considering the similar results in the EPM, it suggests a different scenario. Rats in the control group have made more entries both in the center and at the periphery. Similarly, in the EPM, they have made more entries into both closed and open arms. This indicates a general decrease in movement in the groups with STZ administration compared to the control group.

To clarify this situation, two more parameters are considered: the time spent in the center and the periphery of the open field. In the OFT, rats in the control and S200 groups spent longer durations in the center. Although statistical significance has not been detected, it has been noted that STZ administration decreases the time spent in the center of the open field. A decrease in the time spent in the open field is an indicator of anxiety‐like behavior. Liu, Chen, et al. (2021) observed a decrease in the number of entries and time spent in the center area in MPTP‐treated mice in the OFT, and with silibinin administration, they observed a significant increase in these parameters compared to the MPTP group.

According to the study by Lee et al. (2020) rats administered with 100 mg/kg of silibinin spent more time in the central area and made more square crossings in the OFT test compared to the other groups. Silibinin reversed the effect of the application that led to immobility in the OFT test. On the other hand, in a study conducted by Liu et al. (2020), silibinin did not result in a significant difference in walking speed and distance covered in the OFT test.

#### 4.3.4 Barnes maze

The results of the Barnes maze, a spatial memory test, indicate that the diabetes group exhibited poorer performance in finding the correct escape box compared to the control group (*p* < .05). However, in the groups treated with silibinin, this situation was observed to be closer to the control group. Especially in the S200 group, the time has significantly decreased compared to the diabetes group. This suggests that the learning impairment caused by STZ could be reversed by silibinin (Figure 5).

Literature studies have shown that silibinin reverses exhaustive exercise‐induced learning and memory loss in rats and reduces apoptosis in hippocampal cells (Liu et al., 2019). Although our study did not conduct a brain tissue analysis, the results of our study align with existing behavioral literature data stating protective effects on memory tests such as the Morris maze or preventing apoptosis (Liu et al., 2020). It is stated in the literature that silibinin exhibits a protective effect in neuronal diseases such as Parkinson's and Alzheimer's (Lee et al., 2020; Liu, Chen, et al., 2021). Silibinin is shown to exert protective effects not only on rodent models but also on human trials (Hussain et al., 2022).

When behavior data are collectively evaluated, it can be concluded that STZ administration disrupted locomotor activity based on the results of the rotarod test in rats. According to data from the Barnes maze, a memory test, it impaired performance related to memory. In tests assessing anxiety‐like behavior, such as the open field and EPM, STZ administration increased anxiety‐like behaviors. On the other hand, silibinin administration partially reversed these negative effects caused by STZ, as observed in the Barnes maze.

## 5 CONCLUSION

In this study, our aim was to assess the effects of silibinin on diabetic hyperglycemia, insulin levels, liver function tests, and behavioral outcomes in a streptozotocin (STZ)‐induced diabetic rat model. The obtained results demonstrate that silibinin exhibits positive effects on diabetic hyperglycemia, insulin levels, mitigating increased liver ALT values, and behavioral parameters such as learning and locomotor activity.

Silibinin targets various pathophysiological mechanisms of diabetes by increasing insulin levels, reducing insulin resistance, and lowering hyperglycemia. To understand the molecular‐level effects of these mechanisms, further investigation focused on intracellular signaling pathways and their impact on insulin receptors is necessary.

In conclusion, this study suggests that silibinin could have positive effects on diabetic rats in terms of hyperglycemia, insulin levels, liver function test parameters, and behaviors such as learning and locomotor activity. However, further research is needed to determine whether these findings are applicable to humans, the dosages and mechanisms of efficacy, as well as the long‐term effects of silibinin.

## AUTHOR CONTRIBUTIONS

**Asli San Dagli Gul**: Conceptualization (equal); data curation (equal); investigation (equal); writing – original draft (equal). **Okan Arihan**: Conceptualization (equal); data curation (equal); formal analysis (equal); investigation (equal); methodology (equal); writing – original draft (equal). **Gulbahar Boyuk Ozcan**: Data curation (equal); formal analysis (equal); investigation (equal); methodology (equal); writing – original draft (equal).

## Data Availability

The authors elect to not share data.
